# 

**Published:** 1888-03

**Authors:** 


					﻿Vol. VIII.
MARCH, 1888.
NO. 3.
IHHE
HOMEOPATHIC pHY^lClAfl,
A Monthly Journal of Homoeopathic Materia Medica
and Clinical Medicine.
“ He is a freeman whom truth maizes free, and all are slaves beside."
“Seek the Truth: come whence it may, cost what it will."
EDITED AND PUBLISHED BY
EDMUND J.	M. t>.,
AND
WALTER M. JANIKS, M. D.
PHILADELPHIA:
No. 1123 SPRUCE STREET.
i.o nt u o it «
ALFRED HEATH & CO., Sole Agents hob Great Britain,
114 Ebury Street, Eaton Square.
IHf'Ttearly Subscription, $2.50, invariably in advance. Single numbers t 95 cts.
Entered at the Philadelphia Post-Office as Second-Class Mail Matter.
				

